# BMSC-Derived Exosomes Alleviate Sepsis-Associated Acute Respiratory Distress Syndrome by Activating the Nrf2 Pathway to Reverse Mitochondrial Dysfunction

**DOI:** 10.1155/2023/7072700

**Published:** 2023-03-31

**Authors:** Zhenzhen Li, Beijie Zheng, Chenchen Liu, Xiang Zhao, Yupeng Zhao, Xiangrui Wang, Lei Hou, Zhongwei Yang

**Affiliations:** ^1^Department of Pharmacy, Zhongshan Hospital Wusong Branch, Fudan University, 200940 Shanghai, China; ^2^Department of Anesthesiology, Renji Hospital, School of Medicine, Shanghai Jiao Tong University, 200127 Shanghai, China; ^3^Department of Anesthesiology, Shanghai General Hospital, Shanghai Jiaotong University School of Medicine, 200081 Shanghai, China; ^4^Department of Surgery of Spine and Spinal Cord, Henan University People's Hospital, Henan Provincial People's Hospital, Henan, Zhengzhou 453003, China; ^5^Department of Anesthesiology and Critical Care Medicine, Shanghai East Hospital, Tongji University School of Medicine, 200120 Shanghai, China; ^6^Shanghai East Hospital Ji'an Hospital, 80 Ji'an South Road, Ji'an City, 343000 Jiangxi Province, China

## Abstract

Type II alveolar epithelial cell (AECII) apoptosis is one of the most vital causes of sepsis-induced acute respiratory distress syndrome (ARDS). Recent evidence has proved that bone mesenchymal stem cell-derived exosomes (BMSC-exos) can effectively reduce sepsis-induced ARDS. However, the function and molecular mechanism of BMSC-exos in sepsis-induced AECII apoptosis remain to be elucidated. In the present study, a more significant number of AECII apoptosis, high mitochondrial fission p-Drp1 protein levels, and low levels of mitochondrial biogenesis-related PGC1*α*, Tfam, and Nrf1 proteins accompanied with ATP content depression were confirmed in AECIIs in response to sepsis. Surprisingly, BMSC-exos successfully recovered mitochondrial biogenesis, including the upregulated expression of PGC1*α*, Tfam, Nrf1 proteins, and ATP contents, and prohibited p-Drp1-mediated mitochondrial fission by promoting Nrf2 expression. However, the aforementioned BMSC-exo reversal of mitochondrial dysfunction in AECIIs can be blocked by Nrf2 inhibitor ML385. Finally, BMSC-exos ameliorated the mortality rate, AECII apoptosis, inflammatory cytokine storm including HMGB1 and IL-6, and pathological lung damage in sepsis mice, which also could be prevented by ML385. These findings reveal a new mechanism of BMSC-exos in reversing mitochondrial dysfunction to alleviate AECII apoptosis, which may provide novel strategies for preventing and treating sepsis-induced ARDS.

## 1. Introduction

Severe pulmonary dysfunction, especially acute respiratory distress syndrome (ARDS), the character of pulmonary alveoli edema, decreased pulmonary compliance, and worsening gas exchange, contributes to the morbidity and mortality of septic patients [1]. Under normal physiological conditions, type II alveolar epithelial cell (AECII) physiological function maintenance needs ample mitochondrial biogenesis to generate adenosine triphosphate (ATP) through mitochondrial oxidative phosphorylation [2]. But in sepsis pathological circumstances, lipopolysaccharide (LPS) disrupts mitochondrial biogenesis, which fails to contribute to energy supply in AECIIs, leading to AECII apoptosis and ARDS [3]. In addition, our previous study found that mitochondrial fission is closely related to AECII apoptosis [4]. Mitochondrial fission leads to mass damaged mitochondria which fails to maintain mitochondrial ATP supplement [5]. Therefore, reversing mitochondrial dysfunction after LPS exposure, including recovery of mitochondrial biogenesis and inhibition of mitochondrial fission, may represent an ideal strategy to protect against sepsis-related AECII apoptosis.

Increasing evidence confirmed that nuclear factor erythroid 2-related factor 2 (Nrf2) activation mediates mitochondrial dysfunction improvement in the sepsis rat model [6, 7]. Briefly, Nrf2 is a translational activation protein that enters the nucleus. It helps increase gene expression of mitochondrial functions and turnover-related protein, including mitochondrial transcription factor A (Tfam), nuclear factor erythroid 2-like 1 (Nrf1), and peroxisome proliferator-activated receptor gamma coactivator-1 alpha (PGC-1*α*), independent of transcriptional regulation of antioxidant and detoxification genes [8, 9]. Furthermore, Nrf2 activation successfully blocks phosphorylation of dynamin-related protein 1- (Drp1-) mediated mitochondrial fission [10]. The traditional treatment programs failed to reverse mitochondrial dysfunction to overcome sepsis-related AECII apoptosis in medical research, and a novel treatment is urgently needed. Thus, a new method was used in the present study to reverse mitochondrial dysfunction in sepsis-related AECII apoptosis via the activation of Nrf2.

Bone marrow mesenchymal stem cell- (BMSC-) based therapies have been confirmed to have promising effects in preclinical studies and phase 1 clinical trials in sepsis-related ARDS [11–13]. Animal studies reported that administration of BMSCs successfully balances the proinflammatory (IL-6) and anti-inflammatory cytokines (IL-10) in the pulmonary microenvironment, which increased the survival rate in animal septic models [14, 15]. Increasing studies found that BMSCs exert biological immunomodulatory and therapeutic effects on alleviating ARDS mainly by secreting its exosomes [16, 17]. Exosomes, with a diameter of 30-150 nm, transfer BMSC contents, such as noncoding RNAs and small proteins, to the receptor cells to recover the injured cell's function. Yang et al. recently reported that BMSC-exos prevent hyperoxia-induced AECII apoptosis *in vitro* [18]. Silva et al. further found that BMSC-exos improve mitochondrial dysfunction in human primary lung epithelial after LPS exposure [19]. It has been confirmed that mesenchymal stem cell-derived exosomes could be considered Nrf2 effective agonists [20, 21]. From this, we hypothesized that BMSC-exos could reduce AECII apoptosis by enhancing the Nrf2 pathway to reverse mitochondrial dysfunction in a sepsis-related ARDS animal model.

## 2. Materials and Methods

### 2.1. Animals

Wild-type male C57BL/6J mice aged 6-8 weeks (Jiesijie Experimental Animal Company, Shanghai, China) were housed in a specific pathogen-free environment (12 h light/dark, 22-24°C) and had free access to water and food in Shanghai General Hospital Animal Laboratory. All animal experiments were approved by the Ethics Committee of Shanghai General Hospital (2022AWS0104).

### 2.2. Isolation, Culture, and Differentiation Assays of BMSCs

The primary murine BMSCs were isolated as the previous article described [22]. Briefly, mice were sacrificed by cervical dislocation, and the bone marrow in the femur and tibias was extracted with a 22-gauge syringe. The extracted bone marrow cells were incubated in DMEM/F12 (Gibco, Grand Island, NY) with 12% fetal bovine serum (FBS; Gibco) containing no exosomes and placed at 37°C in a humidified incubator containing 5% CO_2_. After 3 days of incubation, the medium was changed, and nonadherent cells were removed. When the adherent cells reached 90% confluence, the cells were passaged at 1 : 3 ratios. BMSCs at 3-5 generations were employed for all experiments.

The BMSCs were divided into osteogenic, adipogenic, and chondrogenesis differentiation using the Alizarin red S staining, oil red O staining, or Alcian blue staining (Sigma-Aldrich) according to their corresponding Sigma-Aldrich, St. Louis, MO, company instructions.

### 2.3. BMSC-Exo Isolation, Identification, and Labeling

The cell supernatants from 3 to 5 generations of BMSCs were extracted to exosomes using the Exosome Isolation Reagent Kits (#4478359; Thermo Fisher Scientific, Waltham, MA, USA), and the protein concentrations in BMSC-exos were detected with BCA assay (Thermo Scientific, USA). The isolated exosome morphology, size distribution, and surface marker expression (CD63, TSG101) were identified using the transmission electron microscope, nanoparticle tracking analysis, and western blot. Finally, the isolated BMSC-exos were labeled with PKH67 fluorescent dye according to the Sigma-Aldrich, St Louis, MO, manufacturer's instructions. The labeled PKH67 BMSC-exos were cocultured with AECIIs for 24 h incubation and then observed by laser SP8 confocal microscopy.

### 2.4. Cell Culture, LPS Intervention, and BMSC-Exo Treatment

The murine alveolar epithelial cell lines (MLE-12 cells) were obtained from American Type Culture Collection and cultured with DMEM (Gibco, USA) supplemented with 10% FBS at 37°C and 5% CO_2_ incubator. The MLE-12 cells were treated with LPS (Sigma-Aldrich, Louis, MO, USA) at 25 *μ*g/ml concentrations for 6 h, 24 h, and 48 h. To further investigate the protective role of BMSC-exos on LPS-treated AECIIs via the Nrf2 pathway, randomly assigned MLE-12 cells were treated with LPS (25 *μ*g/ml) for 1 h and incubated with BMSC-exos (100 *μ*g/ml) for 48 h. In comparison, another group of randomly assigned MLE-12 cells was pretreated with Nrf2 inhibitor ML385 (20 *μ*M; Selleck Chemicals) for 30 min, followed by LPS (25 *μ*g/ml) for 1 h, and subsequently cocultured with BMSC-exos (100 *μ*g/ml) for 48 h as other teams reported [23, 24].

### 2.5. Cecal Ligation and Puncture (CLP), ML385 Intervention, and BMSC-Exo Treatment

The CLP mouse model was prepared as previously described [25]. Briefly, mice were anesthetized and then cut in a 1 cm midline laparotomy. The cecum was exteriorized, ligated, and punctured with an 18-gauge needle to induce sepsis. A small part of the cecal contents was extruded. After that, the abdominal incision closed with sutures. Control animals underwent the same surgical procedures without cecum ligation and puncture.

To investigate the role of BMSC-exos in protecting sepsis-related ARDS, BMSC-exos (300 *μ*g/mouse) were intratracheally injected into wild-type C57BL/6 mice 1 hour before CLP surgery. In comparison, another group of randomly assigned C57BL/6 mice were administered ML385 (30 mg/kg) intraperitoneally 2 hours before CLP surgery and injected intratracheally with BMSC-exos (300 *μ*g/mouse) 1 hour before CLP surgery. All the above mice were sacrificed under narcotism 48 hours postoperatively.

For the survival experiments, the independent cohort of mice (25 mice per group) received the above treatments, and the survival rate of mice was recorded every day for 7 days. The mice were euthanized on day 7 after the survival rate was recorded.

### 2.6. AECII Apoptosis via Flow Cytometry Assay *In Vivo* and *In Vitro*

*In vivo AECII apoptosis assay*: 24 and 48 hours after CLP surgery, primary murine AECIIs from lung tissue samples were separated via flow cytometry according to Gereke et al.'s reported method with modification [26]. Briefly, lung tissues were digested and collected in a lung cell suspension. Then, cell suspensions were stained with biotinylated antibodies against lineage (Lin) markers: anti-CD45 (hematopoietic cells and alveolar macrophages), anti-CD16/32 (alveolar macrophages), anti-CD31 (endothelial cells), anti-Ter119 (erythroid cells), and anti-integrin *β*4 (club cells and distal lung progenitor cells). Streptavidin-PE was used to place all the lineage marker-positive cells in the PE channel, and EpCAM-APC was used to stain all epithelial cells. At the same time, cells were stained with Annexin V and 7-amino-actinomycin D (7-AAD, PE Annexin V Apoptosis Detection Kit I, BD Biosciences) according to the manufacturer's instructions. All samples were acquired on a BD LSRII flow cytometer (BD Biosciences) and analyzed with FlowJo software (v10.0, Tree Star). FSC^hi^ SSC^hi^, singlet, Lin^−^ EpCAM^+^ cells were the primary AECII population. And the purity was assessed by the expression of pro-SP-C, a specific marker for AECII cells. Double-positive (Annexin V/7-AAD) percentage was defined as apoptotic cell percentage.

*In vitro AECII apoptosis assay*: all the MLE-12 cells in the different groups were directly dyed with Annexin V and 7-AAD and then detected with flow cytometry assay. Generally, the percentage of double positive (Annexin V/7-AAD) was defined as apoptotic cell percentage.

### 2.7. Histopathological and Immunohistochemical Analysis

The pulmonary right upper lobe in different groups was fixed in 10% buffered formaldehyde solution for 24 h and then embedded in paraffin. The tissues were cut into 5 *μ*m sections and, respectively, stained with hematoxylin and eosin (HE) and terminal deoxynucleotidyl transferase dUTP nick end labeling (TUNEL) according to their corresponding instructions (Beyotime Biotechnology, China). The tissue sections were stained with primary antibody HMGB1 (1 : 500 Abcam) for 14 h at 4°C. Next, after being washed with PBS three times, sections were incubated with a secondary antibody, followed by an avidin-biotin-immunoperoxidase and 3,3-diaminobenzidine tetrahydrochloride (DAB). The percentage of stained brown cells, meaning apoptotic cells, was calculated by the number of positively stained brown cells/the total number of cells per microscopic field. A pathologist evaluated the histopathological section's score in a blinded manner according to our previous study method [27]. The intensity of HMGB1 staining was scored with Image-Pro plus 6.0.

### 2.8. Western Blot Assay

Western blot analysis was performed as our article described previously [4]. Total proteins from the BMSCs, MLE-12 cells, and lung tissues were lysed by the RIPA buffer (Beyotime, P0013B), and protein levels were measured with a BCA assay. The protein lysates (30 *μ*g protein) were separated by 8%-12% SDS-PAGE and transferred onto the PVDF membrane. The membranes were blocked with 5% nonfat milk. After washing with TBST three times, the membranes were incubated with anti-CD63 antibody (1 : 1000; CST, USA), anti-TSG101 antibody (1 : 1000; CST, USA), anti- Nrf1 antibody (1 : 1000; Abcam, USA), anti-Tfam antibody (1 : 1000; Abcam, USA), anti-PGC-1*α* antibody (1 : 1000; Abcam, USA), anti-p-Drp1 antibody (1 : 1000; CST, USA), and anti-*β*-actin antibody (1 : 10000; CST, USA) overnight at 4°C. After washing with TBST, the membranes were again incubated with a secondary antibody for 1 h at room temperature. Finally, the membranes were visualized with a chemiluminescence system. The expression of proteins was normalized to *β*-actin as a reference.

### 2.9. Mitochondrial Morphology Fluorescence Staining

The MLE-12 cells in different groups were stained with 200 nM MitoTracker Green FM (Invitrogen, Carlsbad, CA, USA) for 30 min and fixed in 4% paraformaldehyde for 15 min; finally, the cellular mitochondrial morphology was directly observed with the Leica SP8 confocal microscope. The length of mitochondria was evaluated by the Leica SP8 software.

### 2.10. Total Mitochondrial ATP Content

According to the manufacturer's instructions, total mitochondrial ATP contents in MLE-12 cells were examined using an ATP fluorometric assay kit (Beyotime, China). Total ATP levels were expressed as nanomole per milligram of protein.

### 2.11. Total Protein Concentration and Inflammatory Cytokine Levels in Bronchoalveolar Lavage Fluid

The bronchoalveolar lavage fluids (BALF) from all the sacrificed mice were collected as our article described previously [28]. The supernatants in the BALF were detected with IL-6 levels using an ELISA kit under the manufacturer's instructions (R&D Systems, Minneapolis, USA). Total protein concentrations in the supernatants were quantified with a BCA Protein Assay Kit (Thermo Fisher Scientific, Waltham, USA).

### 2.12. Statistical Analysis

Statistics were analyzed using Prism 7.0 (GraphPad Software). All data are presented as means ± standard deviation (SD). In all experiments, an unpaired two-tailed Student *t*-test for two groups or one-way analysis of variance (ANOVA) with Tukey's *post hoc* test for more than two groups was used to compare experimental groups. *P* < 0.01 was considered statistically significant for all experiments.

## 3. Results

### 3.1. Identification of BMSCs and BMSC-Exos

**Figure 1:**
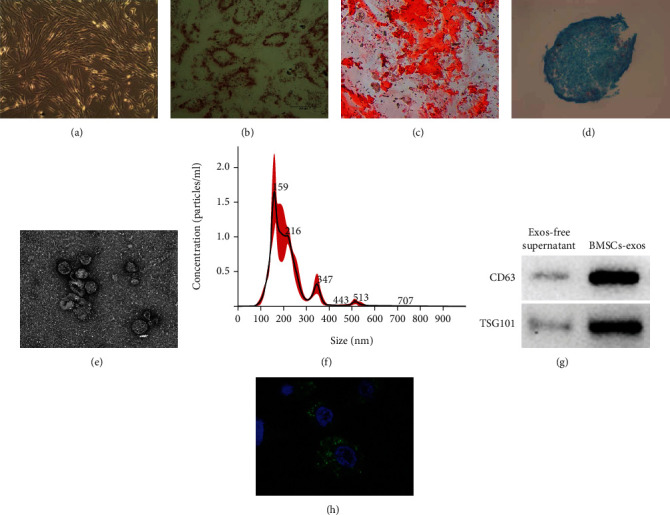
Identification of BMSCs and BMSC-exos. (a) The morphology of BMSCs was observed with an inverted microscope. (b–d) The multidifferentiation potential of BMSCs was identified, respectively, with Alizarin red S staining (b), oil red O staining (c), or Alcian blue staining (d) in vitro. (e–h) The extracted exosomes from BMSCs were further validated by transmission electron microscopy (TEM), nanoparticle tracking analysis (NTA), and western blotting. (e) TEM showed that BMSC-derived exosomes' morphology was circular and cup-shaped (100-200 nm in diameter). (f) NTA showed BMSC-exo size distribution. (g) Western blotting showed that BMSC-exos have specialized markers CD63 and TSG101. (h) Immunofluorescence image showing MLE-12 cells incubated with PKH67-labeled BMSC-exos (green) for 24 hours. Cell nuclei were counterstained with Hoechst (blue).

As shown in Figures 1(a)–1(d), primary BMSCs within 6 to 7 days revealed similar fibroblastic morphologies in shape and could be sequentially differentiated into adipogenic, osteogenic, and chondrogenic lineages under their corresponding differentiation induction. As shown in Figures 1(e)–1(g), supernatant-derived extractions from BMSCs exhibited circular and cup-shaped structures (100-200 nm in diameter), 158.6 nm in the prominent particle size peak, 213.3 nm in the average particle size, and higher CD63 and TSG101 protein levels. All these characterizations suggest that these BMSC-derived extractions from the supernatants were exactly exosomes. In addition, PKH-67-labeled BMSC-exos could reach inside MLE-12 cells (Figure 1(h)).

### 3.2. Sepsis-Subjected AECII Apoptosis Was Closely Positive with Mitochondrial Dysfunction

**Figure 2:**
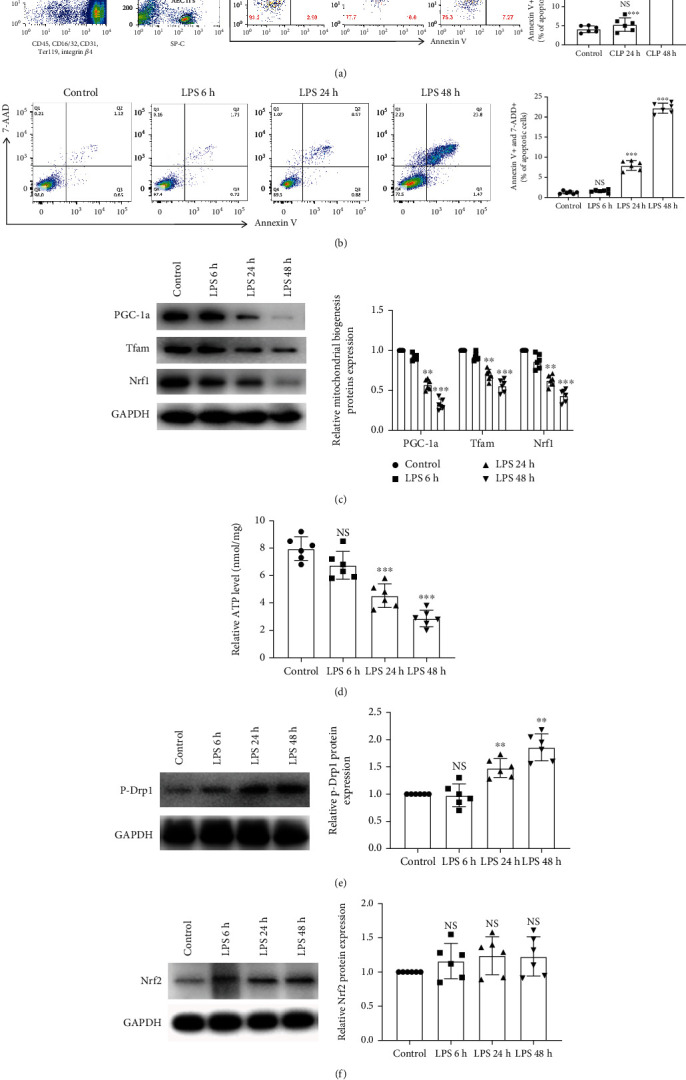
Sepsis-subjected AECII apoptosis was closely positive with mitochondrial dysfunction. (a) The mice were subjected to CLP surgery for 24 and 48 hours. Primary AECIIs were isolated with flow cytometry and stained with Annexin V and 7-AAD. The percentage of double Annexin V- and 7-AAD-positive cells after 48 hours of CLP was almost 16%. (b) The AECIIs (MLE-12 cells) were exposed to LPS (25 *μ*g/ml) for 6, 24, and 48 hours. The percentage of double Annexin V- and 7-AAD-positive cells was detected by flow cytometry. (c) The expression of mitochondrial biogenesis-related proteins (PGC-1*α*, Tfam, and Nrf1) was detected with western blotting. (d) The mitochondrial ATP content was detected by an ATP fluorometric assay kit. (e, f) Mitochondrial fission protein p-Drp1 and antioxidant Nrf2 protein expressions were detected using western blotting. Data are represented as mean ± SD, *n* = 6 per group. NS: nonsignificance; ^∗∗^*P* < 0.01 or ^∗∗∗^*P* < 0.001 vs. the control group.

To better investigate the effects of sepsis on the primary AECIIs, we directly used flow cytometry to isolate AECIIs from mice in sham surgery and CLP surgery (postoperative 24 h, 48 h) and found that sepsis contributed largely to AECII apoptosis (15%) in 48 hours after CLP surgery mice (Figure 2(a)). Next, we examined the effects of LPS exposure on AECII apoptosis and mitochondrial function. MLE-12 cells were treated with 25 *μ*g/ml LPS for 6, 24, and 48 h, respectively. A large proportion of AECII apoptosis (23%) occurred in LPS exposure at 48 hours (Figure 2(b)). The mitochondrial biogenesis PGC-1*α*, Tfam, and Nrf1 protein levels and ATP contents were gradually reduced in LPS-treated AECIIs in a time-dependent manner compared with the control group (Figures 2(c) and 2(d)). Differently, the mitochondrial fission p-Drp1 was gradually upregulated in LPS-treated AECIIs (Figure 2(e)). In addition, the Nrf2 protein, closely related to mitochondrial function, was slightly increased, but no statistical differences in AECIIs were observed after LPS exposure to 24 and 48 h (Figure 2(f)). These data indicated that sepsis-subjected AECII apoptosis was closely positive with mitochondrial dysfunction, especially depressed mitochondrial biogenesis, and extensive mitochondrial fission.

### 3.3. BMSC-Exos Reversed Mitochondrial Dysfunction by Activating the Nrf2 Pathway

**Figure 3:**
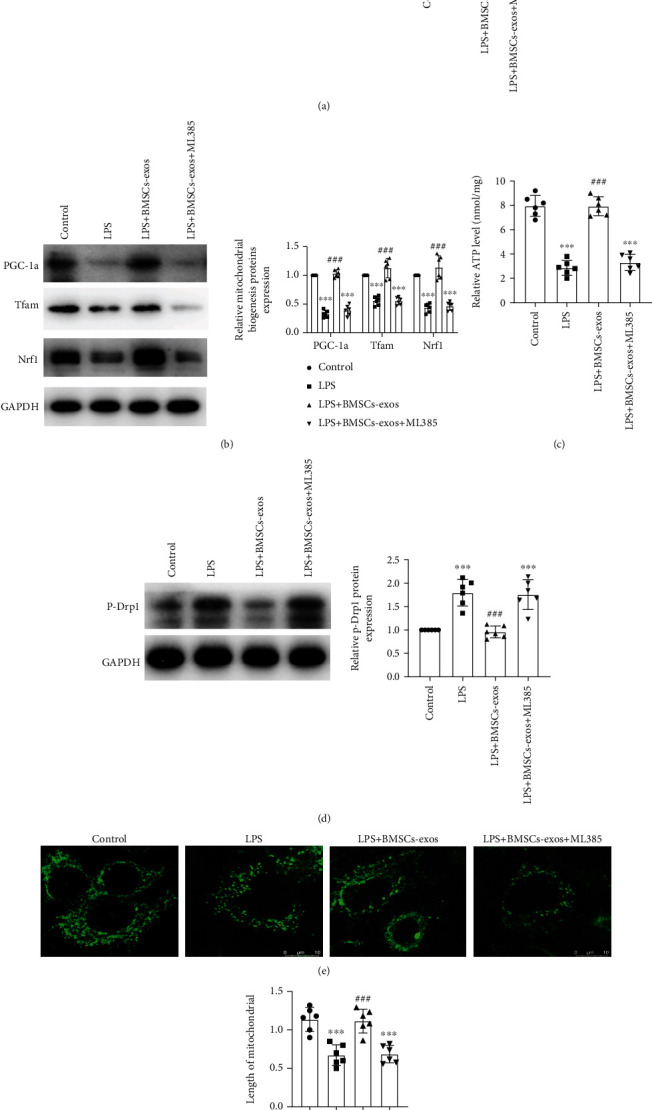
BMSC-exos reversed mitochondrial dysfunction by activating the Nrf2 pathway. AECIIs were pretreated with Nrf2 inhibitor-ML385 (20 *μ*M) for 30 min, followed by LPS (25 *μ*g/ml) for 1 hour, and subsequently cocultured with BMSC-exos (100 *μ*g/ml) for 48 hours. (a, b) Expressions of Nrf2, PGC-1*α*, Nrf1, and Tfam proteins were detected with western blotting. (c) Mitochondrial ATP content in AECIIs was measured with an ATP fluorometric assay kit. (d) Expression of the p-Drp1 protein was detected by western blotting. (e) MitoTracker Green-stained mitochondrial morphology in AECIIs was observed with immunofluorescence. (f) The average length of the mitochondria in each group was analyzed. Data are represented as mean ± SD, *n* = 6 per group. NS: nonsignificance; ^∗∗∗^*P* < 0.001 vs. the control group; ^###^*P* < 0.001 vs. the LPS group.

To determine whether BMSC-exos reversed mitochondrial dysfunction by activating the Nrf2 pathway, we used the Nrf2 inhibitor ML385 to block the role of BMSC-exos on the mitochondrial function of AECIIs. We noticed that ML385 successfully blocked upregulated Nrf2 expression in BMSC-exo- (100 *μ*g/ml) treated AECIIs (Figure 3(a)). Of interest, BMSC-exos promoted Nrf1, PGC-1*α*, and Tfam protein levels (Figure 3(b)) and ATP levels (Figure 3(c)) as well as inhibited p-Drp1 protein levels (Figure 3(d)) in LPS-treated AECIIs, respectively. Importantly, BMSC-exos successfully recover the mitochondrial integrity by comparing fragment mitochondrial in LPS-treated AECIIs (Figures 3(e) and 3(f)). All the above BMSC-exo functions could be blocked with ML385. The above evidence suggested that BMSC-exos reversed mitochondrial dysfunction by activating the Nrf2 pathway.

### 3.4. BMSC-Exos Alleviated Sepsis-Treated AECII Apoptosis via Activating the Nrf2 Pathway In Vivo and In Vitro

**Figure 4:**
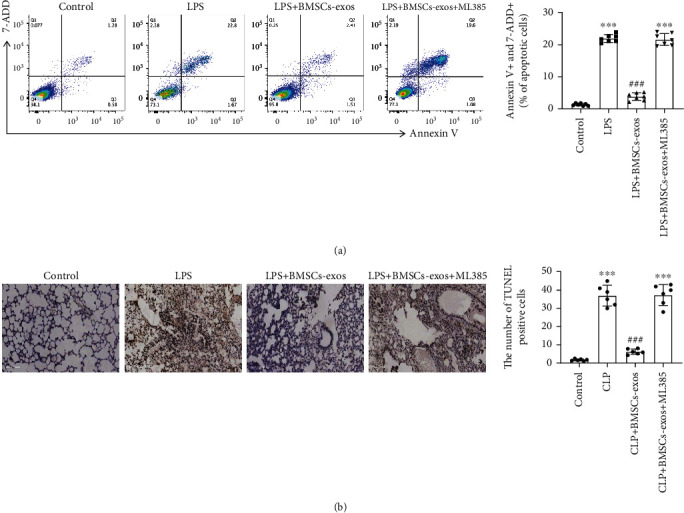
BMSC-exos alleviated sepsis-treated AECII apoptosis via activating the Nrf2 pathway *in vivo* and *in vitro*. AECIIs were pretreated with ML385 (20 *μ*M) for 30 min, followed by LPS (25 *μ*g/ml) for 1 hour, and subsequently cocultured with BMSC-exos (100 *μ*g/ml) for 48 hours. (a) The frequency of double Annexin V- and 7-AAD-positive AECIIs was characterized by flow cytometry. The mice were intraperitoneally injected with ML385 (30 mg/kg) 2 hours before CLP surgery and BMSC-exosomes (300 *μ*g/mouse) 1 hour before CLP surgery. (b) TUNEL staining (brown) was detected in the lung sections of all the treated mice. Data are represented as the mean ± SD, *n* = 6 per group. NS: nonsignificance; ^∗∗∗^*P* < 0.001 vs. the control group; ^###^*P* < 0.001 vs. the LPS group or CLP group.

Next, we separately detected the effect of BMSC-exos on AECII apoptosis *in vitro* with flow cytometry and *in vivo* with TUNEL staining. Flow cytometry showed that BMSC-exos successfully decreased the proportion of AECII apoptosis from 20% to 3% (Figure 4(a)). Equally, TUNEL staining showed large numbers of positive cell staining in lung sections of sepsis mice. In contrast, BMSC-exos remarkably reduce the positive cell sating ratio in sepsis mice lung sections (Figure 4(b)). The above phenomenon could be reversed with the ML385 application (Figures 4(a) and 4(b)). The results suggest that BMSC-exos alleviated sepsis-treated AECII apoptosis via activating the Nrf2 pathway.

### 3.5. BMSC-Derived Exosomes Ameliorated Sepsis-Induced ARDS and Inflammatory Cytokine Release via Activating the Nrf2 Pathway

**Figure 5:**
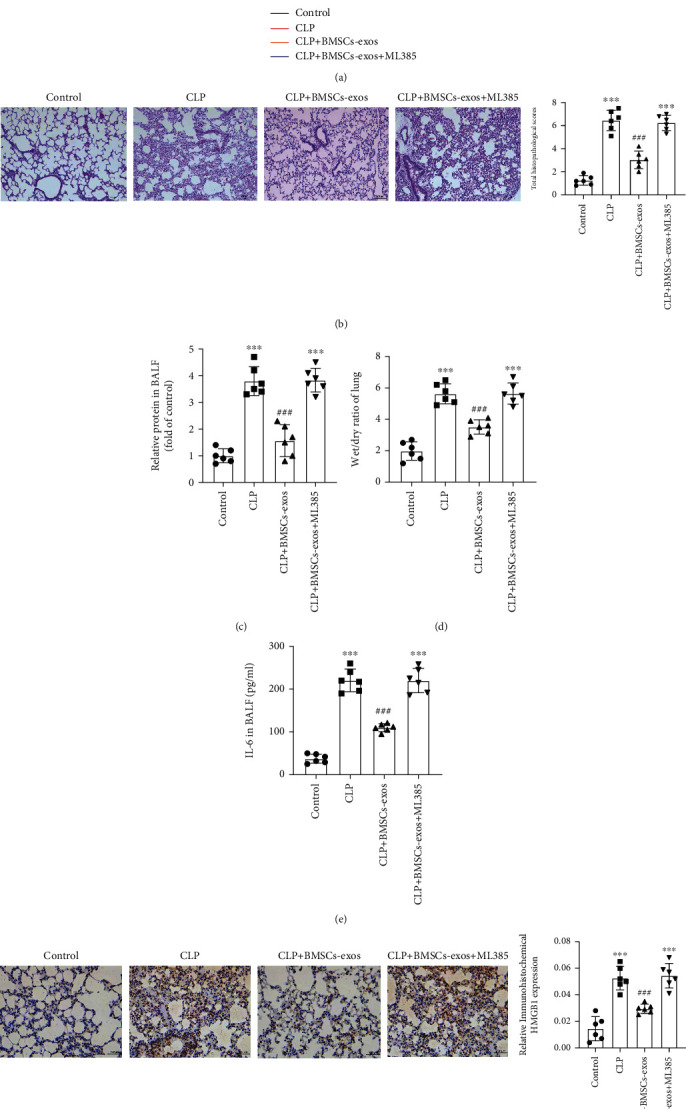
BMSC-exos alleviated sepsis-induced ARDS via activating the Nrf2 pathway *in vivo*. The randomly assigned mice were administered ML385 (30 mg/kg) intraperitoneally 2 hours before CLP surgery and injected intratracheally with BMSC-exos (300 *μ*g/mouse) 1 hour before CLP surgery. (a) Survival rate of mice. (b) H&E staining of lung tissue sections. (c) Total protein concentrations in BALF. (d) Wet/dry ratio of lung tissue. (e) Levels of the IL-6 in BALF. (f) Immunohistochemistry of HMGB1 expression in lung tissue sections. Data are presented as mean ± SD, *n* = 6 per group. NS: nonsignificance; ^∗∗∗^*P* < 0.001 vs. the control group; ^###^*P* < 0.001 vs. the LPS group or CLP group.

Finally, the ARDS animal model was established by CLP mouse surgery to explore the therapeutic potential of BMSC-exos in sepsis-induced ARDS in vivo. Firstly, we observed that BMSC-exos significantly reduced the mortality rate of mice (Figure 5(a)). Histological examinations revealed improvement with alveolar septal thickening, interstitial edema, vascular congestion, and interstitial neutrophil infiltration with the application of BMSC-exos in sepsis-treated mice (Figure 5(b)). Consistently, total protein concentration (Figure 5(c)), wet/dry ratio of the lung (Figure 5(d)), inflammatory cytokine IL-6 (Figure 5(e)) in the BALF, and HMGB1 expression (Figure 5(f)) in the pulmonary tissue significantly decreased. However, BMSC-exos failed to avoid lung tissue damage and reduce mortality by administering ML385 in the sepsis mouse model (Figures 5(a)–5(f)). These results indicated that BMSC-exos could effectively attenuate sepsis-induced ARDS via the Nrf2 pathway.

## 4. Discussions

Although previous studies have demonstrated that mesenchymal stem cell-derived exosomes (MSCs-exos) significantly alleviate sepsis-induced ARDS [29, 30], these studies mainly focus on the protective role of MSCs-exos in regulating macrophage release of inflammatory cytokines. However, the therapeutic effect of BMSC-exos on regulating AECII death and the underlying mechanism of how it functions remain to be determined. In the present study, we found that BMSC-exos alleviated sepsis-induced AECII apoptosis by recovering the Nrf2-mediated mitochondrial dysfunction. Our findings highlight that enhancing the regulatory role of BMSC-exos on AECII mitochondrial function is a potential therapeutic measure to alleviate sepsis-induced ARDS.

AECIIs, as stem cells of the lung epithelium, have vital secretory and regenerative roles in keeping pulmonary microenvironment homeostasis [31]. Many studies have demonstrated that AECII apoptosis is an essential accelerator in developing sepsis-induced ARDS [32, 33]. To better investigate the relationship between AECII apoptosis and sepsis, we directly analyze AECII apoptosis in the CLP-induced sepsis mouse model. And AECII apoptosis was found to cause a significant increase in the development of sepsis. Subsequently, the murine AECII cells (MLE-12) were stimulated with LPS (25 *μ*g/ml) for 6 h, 24 h, and 48 h. Li et al. reported that MLE-12 cell apoptosis was treated with LPS (100 ng/ml) for 24 h [34]. Liu *et al.* found that LPS (500 ng/ml) induced MLE-12 cell apoptosis for 24 h [35]. Consistent with their findings, we found that LPS at 25 *μ*g/ml concentration for 48 h caused MLE-12 cell apoptosis. Therefore, our findings *in vivo* and *in vitro* confirmed that sepsis significantly contributed to AECII apoptosis.

The mitochondrial functions influence the fate of cells [36]. Mitochondrial homeostasis must be maintained to inhibit cell apoptosis by balancing mitochondrial fission and mitochondrial biogenesis [37]. PGC-1*α* is a cotranscriptional regulation factor that induces mitochondrial biogenesis by activating Nrf1, which activates Tfam [38]. The protein Drp1 has been reported to induce anomalous mitochondrial fission, which triggers apoptosis, a death pathway dependent on caspase-3 in various disease models [39, 40] Our results found that mitochondrial biogenesis-related protein levels including PGC-1*α*, Tfam, and Nrf1 and mitochondrial ATP levels were gradually decreased with LPS stimulation, whereas mitochondrial fission p-Drp1 protein was increased. These results suggested that mitochondrial fission and biogenesis might be involved in the pathological process of AECII cell apoptosis. Thus, we speculated that restoration or improving mitochondrial function might be a therapeutic method to resist AECII apoptosis after sepsis stimulation.

The Nrf2 pathway has been identified as having a close relationship with mitochondrial function and cell apoptosis. Increasing evidence confessed that MSC-exos are powerful agonists [21, 41]. Unlike other Nrf2 agonists, MSC-exos have the advantage of not having the toxic side effects of drugs and potentially reaching cells directly within them [42]. The translocation from the cytosol to the nucleus upon hyperactivation and overexpression of Nrf2 protein could regulate target protein expressions as a transcription factor [43]. Our observations favor that the Nrf2 total protein expression in AECs is upregulated after BMSC-exo exposure. Subsequently, the BMSC-exos successfully restored the inhibitory mitochondrial biogenic function by activating the PGC1a, Nrf1, and Tfam proteins. It was reported that the Nrf2 translation from the cytoplasm to the nucleus regulated Nrf1 expression and then PGC1*α* expression [6]. Therefore, we speculated that BMSC-exos might increase the level of Nrf2 in the nucleus. In this study, our results showed that BMSC-exos (100 *μ*g/ml) immediately enter the inside of damaged AECIIs, following significantly promoted Nrf2 protein expression in LPS-treated AECIIs. Besides, BMSC-exos also, respectively, promoted mitochondrial biogenesis and inhibited mitochondrial fission. Importantly, BMSC-exos significantly alleviated AECII apoptosis *in vivo* and *in vitro*. All the above-mentioned phenomena of BMSC-exos could be blocked with Nrf2 inhibitor ML385. All in all, we confirmed that BMSC-exos alleviated sepsis-treated AECII apoptosis via Nrf2 activation to improve mitochondrial function.

As a limitation, we failed to describe in detail how BMSC-exos promote Nrf2 protein expression in AECIIs. MSC-exos contain large numbers of noncoding RNAs, including microRNAs, long noncoding RNAs, or circular RNAs, which regulate target protein expression on its receptor cells [44, 45]. Others found that miR-200a-3p directly inhibits Nrf2 expression with its corresponding 3′ untranslated regions [46, 47]. We surprisingly found that LPS increased miR-200a-3p expression in MLE-12 cells. Moreover, the increased expression of miR-200a-3p was inhibited by BMSC-exos (Supplementary Data, Figure S1), which may contribute to the underlying mechanism and still needs further research. Up to now, these studies found that circRNAs such as circSCMH1 [48], circBACH1 [49], and circST6GALNAC6 [50] and lncRNAs such as lncRNA Gpr137b [51], lncRNA MALAT1 [52], lncRNA SNHG10 [53], and lncRNA TUG1 [54] could reduce the miR-200a-3p expression. In our future research, we will conduct RNA sequencing of BMSC-exos and find which circRNAs or lncRNAs cause miR-200a-3p degradation.

HMGB1, a damage-associated molecular pattern (DAMP), was involved in the pathogenesis of ARDS [55]. We noted that the HMGB1 expression in the lung tissues increased in sepsis, which could be decreased by BMSC-exos. Most importantly, ML385 successfully blocked the BMSC-exos' role of alleviating the HMGB1 expression in the lung tissues, since HMGB1 expression and secretion could lead to AECII apoptosis and macrophage inflammatory response via its receptor-advanced glycation end products and TLR4 [56, 57]. Besides, BMSC-exos successfully alleviated IL-6 levels in BALF serum which could be reversed with ML385. It is logical to speculate that BMSC-exos activate Nrf2, which inhibits AECII apoptosis and inflammatory cytokine IL-6 by blocking HMGB1 expression. Most importantly, our results indicated that BMSC-exos could alleviate sepsis-evoked pulmonary pathologic changes that characterize ARDS [58], all of which could be reversed with ML385.

## 5. Conclusions

**Figure 6:**
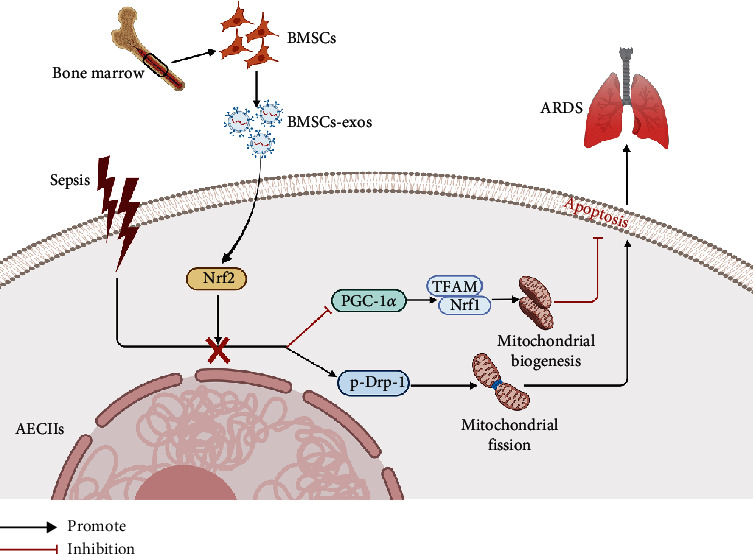
Proposed mechanism for the function of BMSC-exos in alleviating sepsis-associated acute respiratory distress syndrome by activating the Nrf2 pathway to reverse mitochondrial dysfunction. Mitochondrial dysfunction, including depression of mitochondrial biogenesis and excessive mitochondrial fission, might contribute to sepsis-induced AECII apoptosis. BMSC-exos enhanced the expression of Nrf2, subsequently promoting mitochondrial biogenesis and inhibiting mitochondrial fission in AECIIs, alleviating AECII apoptosis and ARDS. Arrows indicate positive regulation, T-bars indicate negative regulation, and cross shows the blocking effect.

Based on this study, we propose a mechanistic model for BMSC-exos alleviating AECII apoptosis in ARDS (Figure 6). The present study demonstrated that BMSC-exos could alleviate sepsis-evoked ARDS by activating the Nrf2 pathway to regulate mitochondrial function. BMSC-exos are a potential therapeutic agent to protect the AECII apoptosis and inflammatory cytokine release following LPS exposure. More research will be entailed to explore the translational medicine application of BMSC-exos for preventing pulmonary tissue damages in clinical patients.

## Data Availability

The data presented in this study are available on request from the corresponding authors. The data are not publicly available due to privacy.

## Supplementary Materials

Supplementary Materials Figure S1: BMSC-exos reversed the sepsis-induced prominent expression of miR-200a-3p. (A) The MLE-12 cell lines were treated with LPS (25 *μ*g/ml) for 6 h, 24 h, and 48 h. To investigate the role of miR-200a-3p in the pathogenesis of sepsis, real-time PCR was used to detect miR-200a-3p levels in LPS-treated MLE-12 cells. We found that the level of miR-200a-3p increased significantly at 6 h and 48 h after LPS treatment compared to the control group. (B) The MLE-12 cell lines were treated with LPS (25 *μ*g/ml) with or without BMSC-exos (100 *μ*g/ml) for 48 h. The expression of miR-200a-3p was detected by real-time PCR. Data are represented as the mean ± SD, *n* = 6 per group. ^∗^*P* < 0.05 and ^∗∗^*P* < 0.01 vs. the control group. Additionally, we examined the effect of BMSC-exos on miR-200a-3p levels at 48 h in LPS-treated MLE-12 cells. Interestingly, BMSC-exos restored the LPS-induced increase of miR-200a-3p at 48 h.
