# Supplementary figures and images for: BMSC-Derived Exosomes Alleviate Sepsis-Associated Acute Respiratory Distress Syndrome by Activating the Nrf2 Pathway to Reverse Mitochondrial Dysfunction
Source: Stem Cells Int. 2023 Mar 31;2023:7072700. doi: 10.1155/2023/7072700 (PMC10081904; doi:10.1155/2023/7072700)

A

Relative mRNA expression of  
miR-200a-3p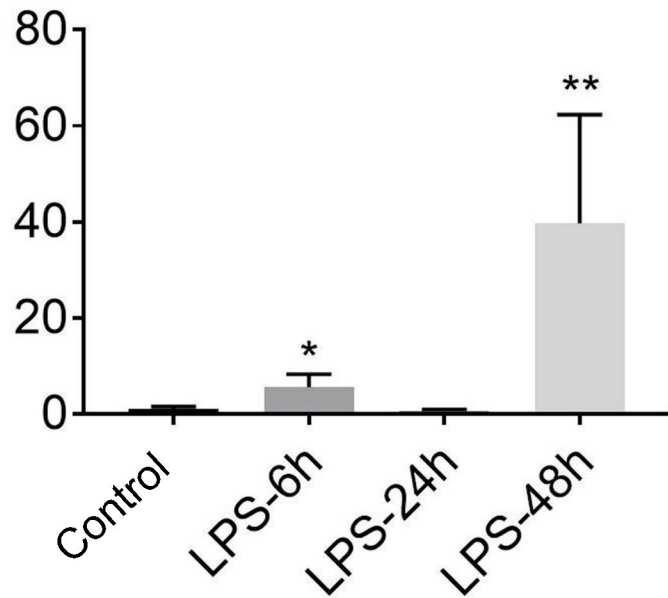

B

Relative mRNA expression of  
miR-200a-3p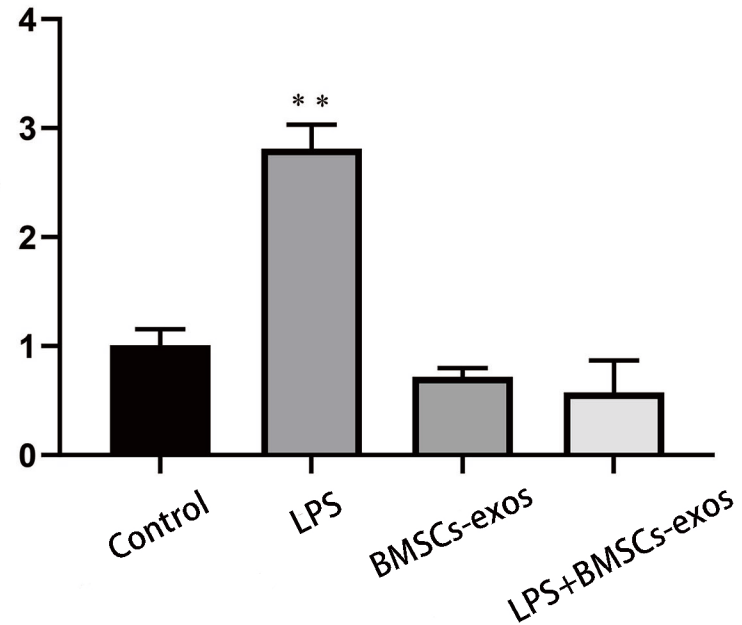

Supplement: Supplementary Materials — Figure S1: BMSC-exos reversed the sepsis-induced prominent expression of miR-200a-3p. (A) The MLE-12 cell lines were treated with LPS (25 μg/ml) for 6 h, 24 h, and 48 h. To investigate the role of miR-200a-3p in the pathogenesis of sepsis, real-time PCR was used to detect miR-200a-3p levels in LPS-treated MLE-12 cells. We found that the level of miR-200a-3p increased significantly at 6 h and 48 h after LPS treatment compared to the control group. (B) The MLE-12 cell lines were treated with LPS (25 μg/ml) with or without BMSC-exos (100 μg/ml) for 48 h. The expression of miR-200a-3p was detected by real-time PCR. Data are represented as the mean ± SD, n = 6 per group. ∗P < 0.05 and ∗∗P < 0.01 vs. the control group. Additionally, we examined the effect of BMSC-exos on miR-200a-3p levels at 48 h in LPS-treated MLE-12 cells. Interestingly, BMSC-exos restored the LPS-induced increase of miR-200a-3p at 48 h. [file 7072700.f1.pdf]
